# Construction of a High-Density Genetic Map Based on SLAF Markers and QTL Analysis of Leaf Size in Rice

**DOI:** 10.3389/fpls.2020.01143

**Published:** 2020-07-31

**Authors:** Yi Wen, Yunxia Fang, Peng Hu, Yiqing Tan, Yueying Wang, Linlin Hou, Xuemei Deng, Hao Wu, Lixin Zhu, Li Zhu, Guang Chen, Dali Zeng, Longbiao Guo, Guangheng Zhang, Zhenyu Gao, Guojun Dong, Deyong Ren, Lan Shen, Qiang Zhang, Dawei Xue, Qian Qian, Jiang Hu

**Affiliations:** ^1^State Key Laboratory of Rice Biology, China National Rice Research Institute, Hangzhou, China; ^2^Rice Research Institute of Shenyang Agricultural University/Key Laboratory of Northern Japonica Rice Genetics and Breeding, Ministry of Education and Liaoning Province, Shenyang, China; ^3^College of Life and Environmental Sciences, Hangzhou Normal University, Hangzhou, China

**Keywords:** rice, leaf, quantitative trait loci, genetic map, specific length amplified fragment sequencing, fine mapping

## Abstract

Leaf shape is an important agronomic trait for constructing an ideal plant type in rice, and high-density genetic map is facilitative in improving accuracy and efficiency for quantitative trait loci (QTL) analysis of leaf trait. In this study, a high-density genetic map contained 10,760 specific length amplified fragment sequencing (SLAF) markers was established based on 149 recombinant inbred lines (RILs) derived from the cross between Rekuangeng (RKG) and Taizhong1 (TN1), which exhibited 1,613.59 cM map distance with an average interval of 0.17 cM. A total of 24 QTLs were detected and explained the phenotypic variance ranged from 9% to 33.8% related to the leaf morphology across two areas. Among them, one uncloned major QTL *qTLLW1* (*qTLL1* and *qTLLW1*) involved in regulating leaf length and leaf width with max 33.8% and 22.5% phenotypic variance respectively was located on chromosome 1, and another major locus *qTLW4* affecting leaf width accounted for max 25.3% phenotypic variance was mapped on chromosome 4. Fine mapping and qRT-PCR expression analysis indicated that *qTLW4* may be allelic to *NAL1* (*Narrow leaf 1*) gene.

## Introduction

Most important agronomic traits are quantitatively inherited, and generally controlled or affected by multiple quantitative trait loci (QTL). Previous studies indicated that high-density genetic map has contributed to the improvement of efficiency and precision for QTL analysis (Yu et al., 2011; Gao et al., 2013). So, a genetic linkage map, especially a high-density genetic map is essential for QTL mapping. There are several types of molecular markers have been used to construct genetic maps during the past 30 years, such as RFLP, AFLP, RAPD, SSR, STS, etc (McCouch et al., 1988; Song and Gustafson, 1995; Yoshimura et al., 1995; Prins et al., 2001; McCouch et al., 2002), but these developed markers are difficult to meet the requirement of high-density genetic map due to low density of polymorphism over the whole genome. Excitingly, the emergence of massive single nucleotide polymorphism (SNP) produced by high-throughput genotyping and sequencing method has well solved this problem, which is suitable to build a high-density genetic linkage map to ensure the veracity of QTL mapping, especially in the species of large size genome and fewer polymorphisms (Huang et al., 2009; Liu et al., 2014; Zhang J. et al., 2015).

Specific-locus amplified fragment sequencing (SLAF-seq) is a strategy based on reduced-representation genome sequencing technology (Sun et al., 2013). Through sequencing of SLAF-seq library constructed by genomic DNA of special enzyme digestion, the polymorphic SLAFs obtained from difference sequence analysis. Among them, each SLAF locus corresponds to a specific fragment generated by enzyme digestion, which includes difference sites of insertion-deletion (InDel) and SNP (Sun et al., 2013). The technology enables us to obtain enough polymorphic markers to carry out QTL mapping and marker-assisted selection (MAS), and exhibits several advantages than conventional molecular markers, including rich polymorphism, uniform distribution, avoiding repeated sequences, not missing important regions of chromosome segment, low cost, short cycle and so on (Sun et al., 2013; Chang et al., 2018). Therefore, SLAF-seq has been widely and successfully applied for genetic map construction and QTL analysis in multiple species (Chen et al., 2013; Li et al., 2014; Zhu et al., 2016; Yin et al., 2018; Zhu et al., 2018; Wu et al., 2019; Yu et al., 2019).

Rice is a worldwide food crop and plays a vital role in eliminating hunger. After application of green revolution gene *sd1* and utilization of hybrid heterosis, breeding of ideal plant architecture for constructing high-quality rice varieties has been increasingly concerned (Jiao et al., 2010; Yuan, 2011). As an essential part of ideal plant architecture, leaf plays an important role in plant growth and development attributing to the function of photosynthetic and respiratory organ. The appropriate leaf shape and upstanding extension posture ensure the high photosynthetic efficiency, which has a decisive effect on energy distribution and carbohydrate accumulation. Among them, the top three leaves are the main source of carbohydrates, which significantly affects the rice yield (Yan et al., 2008; Du et al., 2018). So, a number of genes and QTLs related to regulating leaf morphology have been identified and located, which laid a solid foundation for molecular breeding to improve plant type in rice (Dong et al., 2004; Hu et al., 2010; Wang et al., 2012; Zhang et al., 2014; Zhang B. et al., 2015; Yin et al., 2017; Tang et al., 2018).

In this study, we obtained 63,538 effective SLAFs genotyped in 149 recombinant inbred lines (RILs) and a high-density genetic linkage map containing 10,760 SLAF markers on 12 chromosomes was constructed, which spanned 1,613.59 cM with 0.17 cM interval on average. The genetic map was employed to identify leaf morphology QTLs, and total of 24 QTLs were detected. Of which, two major loci were identified, one locus *qTLLW1* that regulated both leaf length and width explained max 33.8% and 22.5% phenotypic variance, respectively, and another *qTLW4* only affected leaf width accounted for max 25.3% phenotypic variance. Using fine mapping approach, the *qTLW4* was narrowed to the interval of 62.5kb in which contained the *NAL1* gene (Qi et al., 2008). Expression analysis showed that the wide-leaf lines were significantly higher that narrow-leaf lines in RILs, suggesting that *qTLW4* may be an allelic gene of *NAL1*.

## Materials and Methods

### Plant Materials, Trait Measurement, and DNA Extraction

A set of 149 RILs of F_8_ generation derived from the cross of *japonica* cultivar Rekuangeng (RKG) and *indica* cultivar Taizhong1 (TN1) was applied for genetic map construction and QTL analysis. The RIL population had a stable heredity and self-cultivated for many years at China Rice Research Institute, Hangzhou, Zhejiang China (119°950E, 30°070N) and Lingshui, Hainan, China (110°020E, 18°480N). The third leaf length and width of main stem were measured at the full heading stage in Lingshui in 2017 and Hangzhou in 2017, 2018, respectively, and measurement takes three repetitions per line to ensure data accuracy. Data correlation analysis was conducted by using SPSS 19.0 Statistics. The leaf blades of 149 RILs and two parents were collected at heading stage, and CTAB method was used to extract genome DNA for sequencing and genotyping.

### SLAF Library Construction and High-Throughput Sequencing

SLAF library construction was performed following the description by Sun et al. (2013) and Li et al. (2014) with little alteration. The 382Mb rice (*Oryza sativa*) genome was selected as a reference genome to perform a pilot experiment for evaluating efficiency of restriction enzyme digestion and size of restriction fragments. According to the optimal enzyme digestion protocol summarized from pilot experiment, genome DNA of RILs and parents were finally digested by *HaeIII* and *Hpy166II* (NEB) restriction enzyme. After a series of PCR reaction, adapter ligation reactions and agarose gel purification, the production of 264-314bp fragments were isolated and subjected to PCR amplification following the guide of Illumine sample preparation. The sequencing was performed using the IlluminaHiSeq™ 2500 platform (Illumina, Inc; San Diego, CA, USA) at Biomarker Technologies Corporation (Beijing, China).

### SLAF-Seq Data Grouping and Genotyping

SLAF-seq data grouping and genotyping were conducted following the description from Sun et al. (2013), and a desired cluster density would ensure that SLAFs corresponding with map requirements. The raw sequencing data were identified by Dual-index paired-end sequencing approach (Kozich et al., 2013) to acquire effective original reads. Then, using the Burrows-Wheeler Alignment (BWA) software (Li and Durbin, 2009), all the reads were mapped to the reference genome (*Oryza sativa L*.) and SLAFs were developed from the parental cultivars and RILs. Among them, identical reads with 90% similar sequences were considered as one SLAF locus to avoid computational repetition (Kent, 2002). Polymorphism analysis was performed based on the difference between the number of alleles and the sequence of the gene, and SLAFs with polymorphic sites (mainly include SNPs and InDels) were selected for subsequent analysis. Following the general two-element genotype coding rules of genetics, polymorphic SLAFs were then encoded into eight segregation patterns according to the genotype of the parental cultivar (**Supplementary Table S1**). Because the RIL population (F_8_) was derived from two fully homozygous parental cultivars with genotype AA and BB, only the SLAFs with AA x BB segregation pattern was valid for succeeding genetic map construction (**Supplementary Table S1**).

### Construction of High-Density Genetic Map and QTL Identification

The obtained high quality SLAF markers were divided into 12 chromosome (Chr) linkage groups and a modified logarithm of odds scores (MLOD) threshold ≥ 5 was set as default (Stam, 1993). HighMap mapping software was employed to convert recombination frequency to centiMorgans (cM) and then a high-density genetic map based on polymorphic SLAF markers was constructed. The collinearity with the rice reference genome and heat map reflecting recombination frequency of SLAF markers were also used to evaluate the quality of genetic map. QTL analysis was performed *via* high-density genetic map using qgene-4.3.10 software (Joehanes and Nelson, 2008). The composite interval mapping (CIM) method was adopted with a walking distance of 1 milliMorgan to cover the genome completely. The logarithm of odds (LOD) threshold ≥ 3 was considered for statistical significance (P = 0.05), and each QTL interval was determined using 1,000 permutations. The candidate genes of fine mapping regions were analyzed and annotated *via* the database of NCBI (http://www.ncbi.nlm.nih.gov/) and Rice Genome Annotation Project (http://rice.plantbiology.msu.edu/).

### RNA Extraction and qRT-PCR

Total RNA was extracted and purified from fresh leaves at heading stage by Axygen’s RNA Miniprep kit. Subsequently, RNA was converted into cDNA using a reverse transcription reagent kit (ReverTra Ace quantitative PCR RT Master Mix) manufactured by TOYOBO Co., Ltd. All the experimental procedures were carried out following the manufacturer’s instructions. The products were diluted with water in the ratio of 1:10 for quantitative reverse transcription PCR (qRT-PCR). In this study, the 2×SYBR Green PCR Master Mix produced by TOYOBO was employed to amplify the cDNA template on Bio-Rad CFX96 Touch Real-time PCR instrument. Using the 2^-ΔΔT^ method, Relative expression values were obtained by standardization with the *Actin* transcription, each target sample was subjected to three biological replications. All values represent means ± SD (n=3), *P < 0.05, **P < 0.01, based on a Student’s *t-*test. All primer sequences were listed in **Supplementary Table S2**.

## Results

### Sequencing and Genotyping Based on SLAF-Seq

**Figure 1:**
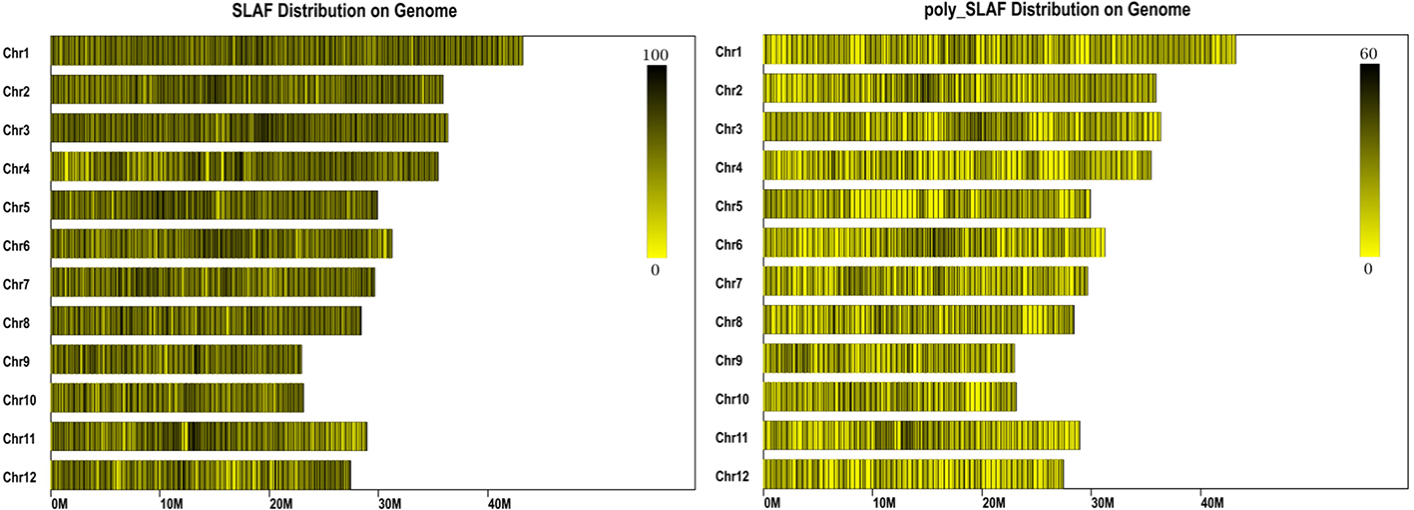
Distribution of specific length amplified fragments (SLAFs) on rice genome. The x-axes indicates chromosome length. Each yellow band represents a chromosome, each black line indicates a SLAF marker.

A total of 47 Gb data containing 211,574,337 reads were obtained by high throughput sequencing, and average Q30 (quality score of 30) and GC (guanine-cytosine) content were 92.54% and 43.54%, respectively (**Table 1**). These reads were then aligned to the reference rice genome *via* BWA software (Li et al., 2009), and the single locus without repetition was considered as a valid SLAF locus. According to the sequence difference, the SLAFs were divided into three types: Polymorphic, Non-Polymorphic, and Repetitive. Among them, 78,374 polymorphic SLAFs were identified and accounted for 32.13% of total 243,955 SLAFs (**Supplementary Table S3**). These SLAFs were evenly distributed on 12 chromosomes (**Figure 1**) and then 63,538 SLAFs were successfully genotyped according to the general two-element genotype coding rules of genetics. Due to fully homozygous of parents with genotype AA or BB, only SLAFs with AA or BB segregation type were useful, and total 59,155 valid polymorphism were obtained and occupied 24.25% (**Supplementary Figure S2**). The Raw sequencing data were upload to the NCBI (https://www.ncbi.nlm.nih.gov/sra/, accession number SRR12043868 - SRR12044018)

**Table 1:** Data statistics for each sample.

Sample ID	Number of Reads	Q30 Percentage (%)	GC Percentage (%)
RKG	11,969,608	92.45	42.8
TN1	12,549,797	92.69	43.06
Offspring	187,054,932	92.47	44.75
Average	/	92.54	43.54
Total	211,574,337	/	/

### Construction of High-Density Genetic Linkage Map for Rice

**Figure 2:**
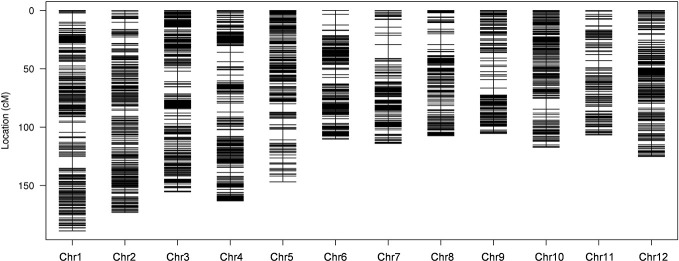
High-density genetic linkage map. The x-and y-axis represent chromosomes and genetic distance, respectively. Each black line indicates a specific length amplified fragment (SLAF) marker.

To ensure a high-quality genetic map, the unqualified markers with the MLOD values below 5 were removed and 10,760 SLAF markers were finally used to construct high-density linkage genetic map by screening 59,155 genotyped SLAFs. The obtained 10,760 SLAF markers were mapped to 12 chromosomes by HighMap software with mean coverage depth 110.77-fold in RKG, 106.72-fold in TN1, and 11.39-fold in RILs. The final genetic linkage map exhibited a total map distance of 1,613.59 cM with the average interval distance of 0.17 cM, and gap<5cM value reached 99.66% (**Figure 2**; **Table 2**). In addition, the average proportion of the valid markers on the map of each individual in the mapping group reached 89.1%, which ensured the accuracy of the map genotyping (**Supplementary Figure S3**). In order to further evaluate the quality of markers linkage relationship, two-dimensional heat maps of 12 chromosomes were separately built, and the results showed the closer distance between adjacent markers came along with a relatively lower recombination frequency, which suggested the precision of the high-density genetic map was reliable (**Supplementary Figure S4**). Collinearity analysis of 12 chromosomes with rice genome was also conducted, and the spearman correlation coefficient between them was close to 1, indicating that most markers on each chromosomes was highly consistent with reference genome and the genetic recombination rate was calculated accurately (**Table 2**; **Supplementary Figure S5**).

**Table 2:** Basic information of high-density genetic map.

ChrGroup ID	TotalSLAFs	TotalDistance (cM)	AverageDistance (cM)	Spearman	Maxgap (cM)	Gap< 5 cM
Chr01	1,222	188.85	0.15	1.00	10.03	99.75%
Chr02	1,529	173.11	0.11	1.00	6.08	99.80%
Chr03	1,201	155.45	0.13	1.00	9.01	99.83%
Chr04	1,002	163.18	0.16	1.00	8.14	99.60%
Chr05	524	146.83	0.28	1.00	9.14	99.43%
Chr06	991	110.31	0.11	0.97	9.19	99.80%
Chr07	423	113.98	0.27	1.00	11.89	98.05%
Chr08	887	107.33	0.12	0.99	9.19	99.77%
Chr09	716	105.44	0.15	1.00	7.28	98.72%
Chr10	1,086	117.24	0.11	1.00	9.32	99.91%
Chr11	378	106.71	0.28	0.97	5.97	95.20%
Chr12	801	125.16	0.16	1.00	4.89	100.00%
Total	10,760	1613.59	/	/	/	/

### Leaf Traits and Data Analysis

**Figure 3:**
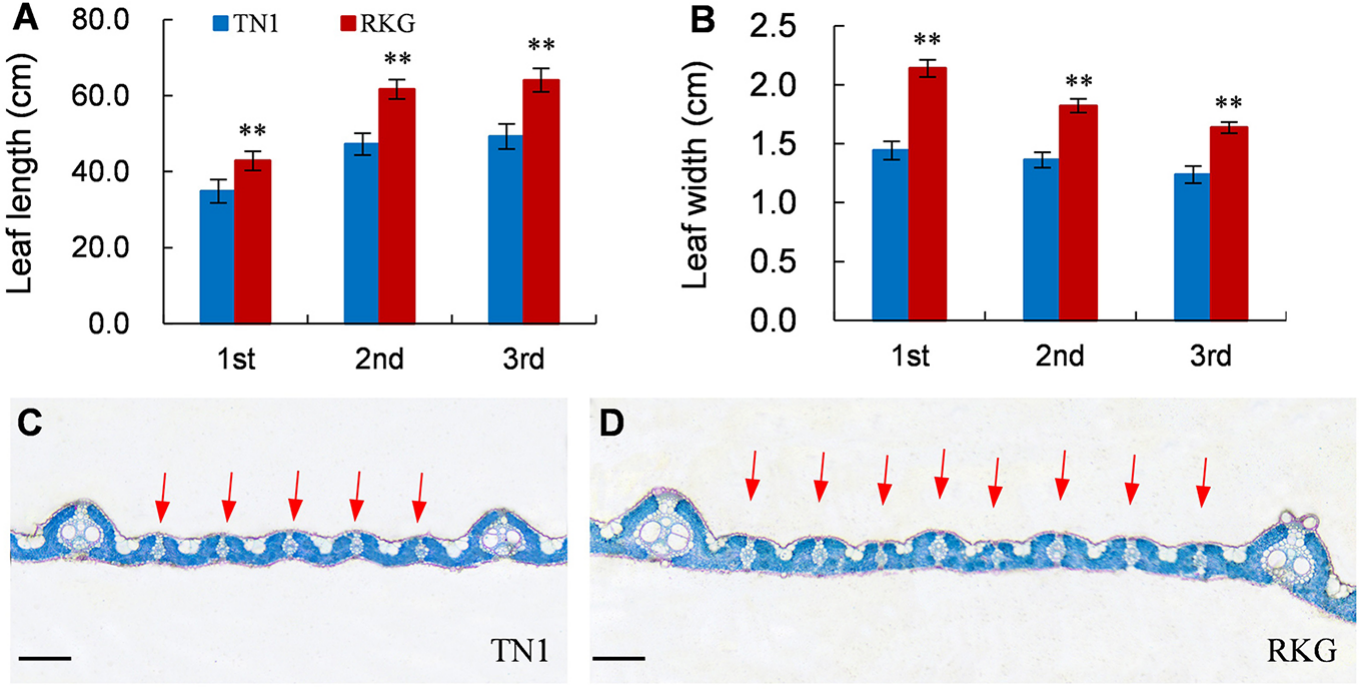
Leaf traits of TN1 and RKG. **(A, B)** Leaf length and width of top-three leaf (n=10). ** indicates a significant difference (P < 0.01) by student’s t-test. **(C, D)** Paraffin cross-section of third leaves, the red arrow indicates the small vein, Scale bar = 20μm.

**Figure 4:**
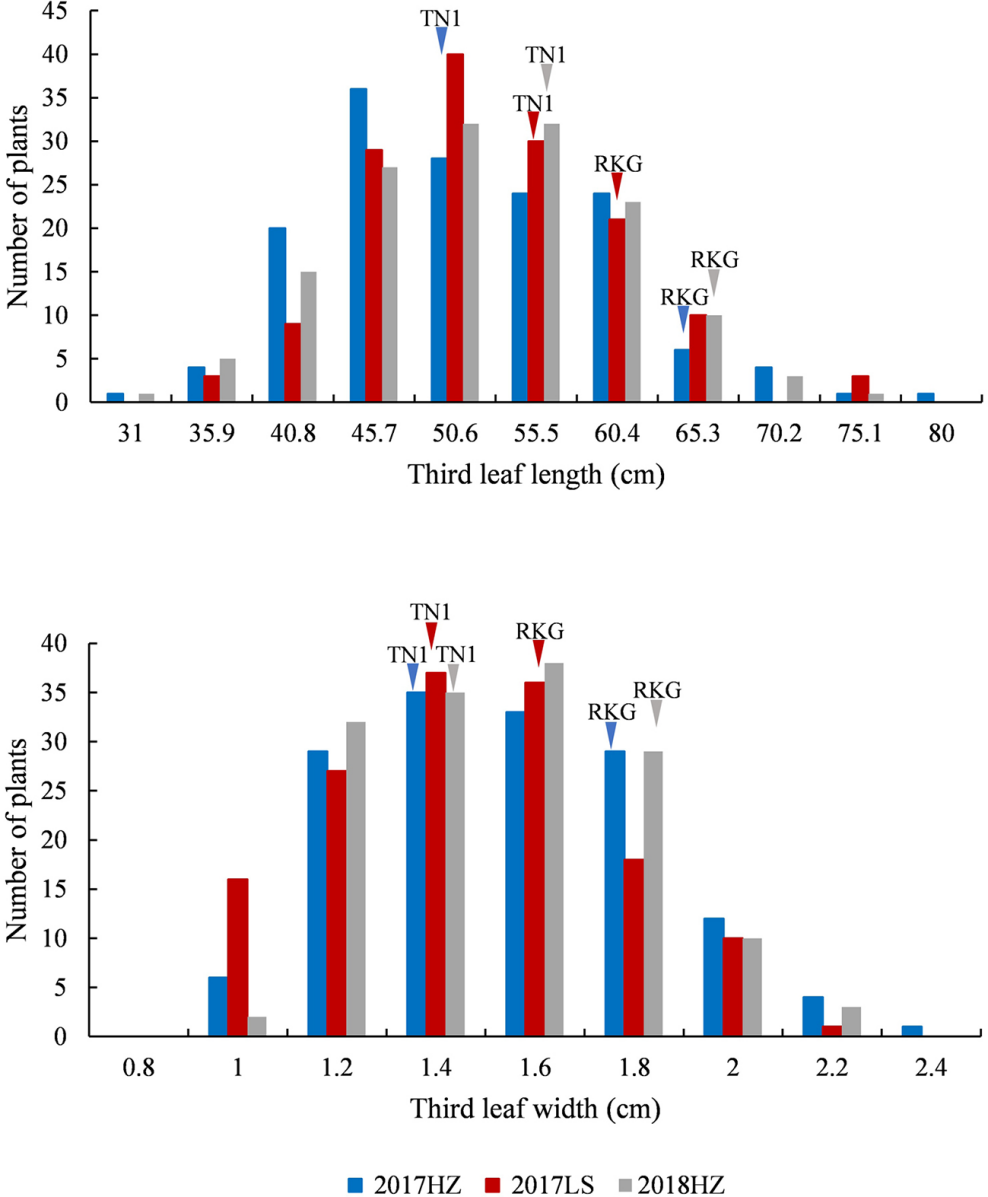
The frequency distribution of third leaf length and width. The x-axis indicates leaf length and leaf width value planted in Lingshui Hainan (LS) in 2017, and Hangzhou Zhejiang (HZ) in 2017 and 2018. The left y-axis indicates the number of individuals, the phenotypic values of the parents (TN1 and RKG) are pointed by arrows, respectively.

At the full heading stage, the leaf length and leaf width of two parent cultivars and 149 RILs were investigated in Lingshui Hainan in 2017 and Hangzhou Zhejiang in 2017, 2018, respectively. The male parent RKG exhibted more longer and wider than female parent TN1 at all top-three leaves, and tissue anatomy analysis also showed that the number of small veins in RKG was significantly more than TN1 (**Figure 3**, **Supplementary Figure S1**). The spearman correlation coefficients between leaf traits of RILs were analyzed by SPSS, and the result indicated that the leaf length versus leaf length and leaf width versus leaf width all exhibted extremely significant positive correlations in both areas, suggesting the correlation traits may be affected by the same gene or have a linkage effect (**Table 3**). The distribution of trait statistics showed a significant differences between the two parents and displayed a continuous segregation values among the 149 individuals with less skewness and kurtosis, which suggested all the phenotype data followed a normal distribution (**Figure 4** and **Table 4**), and were suitable for QTL mapping.

**Table 3:** Spearman correlation analysis of leaf morphology.

Trait	TLL-2017HZ	TLW-2017HZ	TLL-2017LS	TLW-2017LS	TLL-2018HZ	TLW-2018HZ
TLL-2017HZ	1					
TLW-2017HZ	0.039	1				
TLL-2017LS	0.711**	-0.091	1			
TLW-2017LS	-0.047	0.712**	-0.002	1		
TLL-2018HZ	0.859^**^	-0.036	0.743**	-0.097	1	
TLW-2018HZ	0.010	0.789**	-0.049	0.711**	-0.034	1

Data are represented as mean ± SD (n=3). Asterisks represent significant difference determined by Student’s t-test at p-value <0.01(**).

**Table 4:** Descriptive statistic of leaf morphology.

Traits	RKG	TN1	RIL population		Skewnees	Kurtosis
	Mean ± SE (cm)	Mean ± SE (cm)	Range (cm)	Mean ± SE (cm)		
2017HZ-TLL	64.08 ± 3.10	49.25 ± 3.30^**^	31.00-78.23	48.90 ± 8.47	0.586	0.347
2017HZ-TLW	1.64 ± 0.05	1.24 ± 0.07^**^	0.83-2.28	1.45 ± 0.27	0.306	-0.208
2017LS-TLL	58.03 ± 0.56	51.80 ± 1.81^**^	34.37-73.57	50.20 ± 7.32	0.504	0.485
2017LS-TLW	1.55 ± 0.05	1.25 ± 0.05^**^	0.83-2.07	1.38 ± 0.27	0.283	-0.556
2018HZ-TLL	62.53 ± 2.31	51.03 ± 1.51^**^	30.37-71.43	49.65 ± 8.02	0.074	-0.283
2018HZ-TLW	1.74 ± 0.05	1.24 ± 0.06^**^	0.90-2.13	1.44 ± 0.26	0.350	-0.518

Mean ± SE, represents the means and the standard deviation. Asterisks represent significant difference determined by Student’s t-test at p-value < 0.01(**).

### QTL Mapping of Leaf Length and Leaf Width

**Figure 5:**
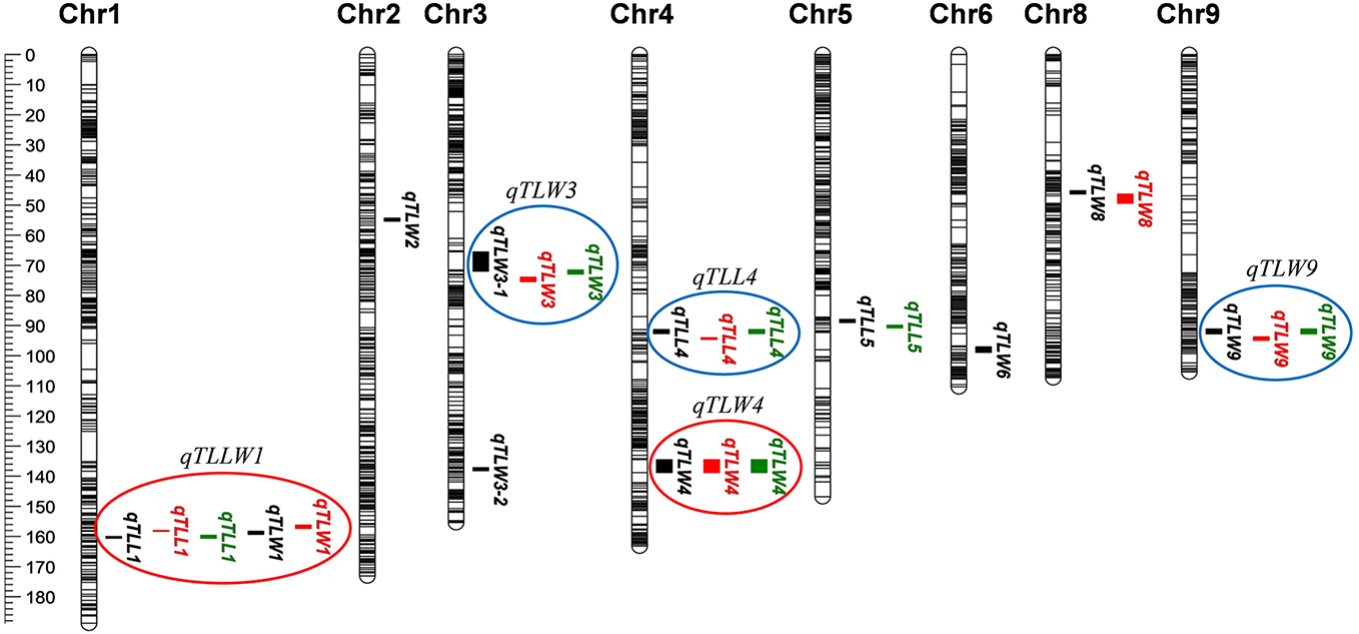
Quantitative trait loci (QTL) analysis of leaf length and width in recombinant inbred lines (RILs). Location of 24 QTLs for leaf length and leaf width. Red circles (major QTLs) and blue circles represent QTLs of repeated detection in both areas, each black line on chromosome indicates a SLAF marker.

Using qgene-4.3.10 software, a total of 24 QTLs underlying leaf morphology were detected on nine chromosomes (1, 2, 3, 4, 5, 6, 8, 9), explained a phenotypic variance ranged from 9% to 33.8% (**Figure 5**, **Table 5** and **Supplementary Figure S6**). Among them, 3 QTLs for leaf length and 8 QTLs for leaf width were detected in Hangzhou in 2017, 2 QTLs for leaf length and 5 QTLs for leaf width were mapped in Lingshui in 2017, and 3 QTLs for leaf length and 3 QTLs for leaf width were located in Hangzhou in 2018. The number of QTLs in 2018 were significantly less than in 2017, but two major QTLs interval with high LOD value and high phenotypic variance were repeatedly detected in both years. In which, 3 leaf width QTLs of third leaf, *qTLW4-2017HZ*, *qTLW4-2017LS*, and *qTLW4-2018HZ* with 11.9%–25.3% phenotypic variance were all located in the same interval between Marker3557229 and Marker4046323. Therefore, they should be controlled by the same QTL, and named *qTLW4* (*Third leaf width on chromosome 4*, **Table 5** and **Figure 5**). In addition, *qTLL1-2017HZ*, *qTLL1-2017LS*, *qTLL1-2018HZ*, *qTLW1-2017HZ*, and *qTLW1-2017LS* were detected in the region of Marker1499909-Marker2090774, the positions of these QTLs were neighboring to each other, and were all located in the region of 1101.7kb. Considering the effects of environmental and measurement errors, we speculated the 5 QTLs were regulated by the same gene, and named it *qTLLW1* (*Third leaf Length and Width on chromosome 1*, **Table 5** and **Figure 5**).

**Table 5:** Leaf length and leaf width quantitative trait locis (QTLs) detected in the TN1×RKG RIL population at full heading stage.

Trait/QTL	Chr	Marker interval	Interval (cM)	LOD value	Additive effect	R^2^(%)
Third leaf length-2017HZ						
*qTLL1*	1	Marker2074344-2090774	159.974–160.632	13.333	4.931	33.8
*qTLL4*	4	Marker3834306-3816055	91.25–92.719	4.684	3.183	13.5
*qTLL5*	5	Marker4136052-4570022	87.865–88.965	4.975	-3.224	14.3
Third leaf length-2017LS						
*qTLL1*	1	Marker1680166-2079436	157.964–158.304	11.321	3.908	29.5
*qTLL4*	4	Marker4055738-3844966	94.133–94.587	4.646	2.673	13.4
Third leaf length-2018HZ						
*qTLL1*	1	Marker1445335-2090774	159.649–160.632	11.357	4.358	29.6
*qTLL4*	4	Marker3834306-3816055	91.25–92.719	3.703	2.69	10.8
*qTLL5*	5	Marker4353359-4552154	89.81–90.826	3.319	-2.564	9.7
Third leaf width-2017HZ						
*qTLW1*	1	Marker1584276-2049210	158.304–159.324	5.995	-0.113	16.9
*qTLW2*	2	Marker3078195-3512446	54.25–55.319	3.532	0.087	10.3
*qTLW3-1*	3	Marker5167535-4966946	65.536–71.862	3.563	0.089	10.4
*qTLW3-2*	3	Marker4874046-4864618	137.172–138.186	3.265	0.084	9.6
*qTLW4*	4	Marker3557229-4046323	134.477–138.848	9.423	0.136	25.3
*qTLW6*	6	Marker55269-501963	97.125–98.949	3.066	-0.082	9
*qTLW8*	8	Marker2444292-2110051	45.168–46.258	3.187	0.083	9.4
*qTLW9*	9	Marker5709498-6016212	91.059–91.792	3.238	0.084	9.5
Third leaf width-2017LS-						
*qTLW1*	1	Marker1499909-1781743	156.188–157.259	8.252	-0.128	22.5
*qTLW3*	3	Marker4892551-4661925	73.933–75.393	3.037	0.084	9
*qTLW4*	4	Marker3557229-4046323	134.477–138.848	4.103	0.095	11.9
*qTLW8*	8	Marker2545129-2208388	46.258–49.357	3.062	0.081	9
*qTLW9*	9	Marker5796105-5946381	93.671–94.905	3.704	0.089	10.8
Third leaf width-2018HZ						
*qTLW3*	3	Marker4716889-5107515	71.507–72.903	3.947	0.088	11.5
*qTLW4*	4	Marker3557229-4046323	134.477–138.848	4.154	0.091	12
*qTLW9*	9	Marker5709498-6016212	91.059–91.792	3.919	0.086	11.4

Positive and negative values indicate additive effect alleles inherited from TN1 or RKG, respectively.

### Fine Mapping of *qTLW4*

**Figure 6:**
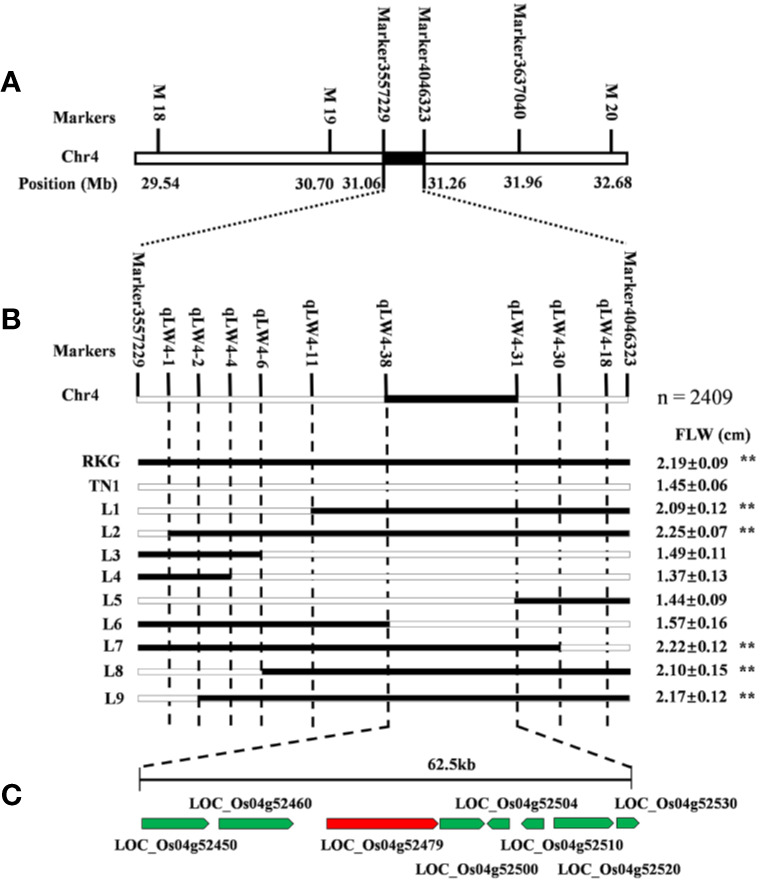
Fine mapping of *qTLW4*. **(A)** Primary map of *qTLW4*. *qTLW4* was primarily located into 194.2 kb. **(B)** Fine-scale map of *qTLW4*. Black and white rectangles indicates homozygous genotype regions for Rekuangeng (RKG) and TN1, respectively. The leave width of corresponding recombinant plants, ** indicates a significant difference (*P* < 0.01) compared to TN1 by the Student’s *t-*test. **(C)** Total eight annotated open reading frames were predicted in the 62.5 kb region.

The major leaf width QTL *qTLW4* was preliminarily located on chromosome 4 between SLAF Marker3557229 and Marker4046323, which contained 194.2 kb physical region (**Figure 6A**). In order to further fine mapping the gene, a BC_2_F_2_ population was constructed using the TN1 as recurrent parent, and a total of 2409 individuals were obtained in paddy field. First, the 186 extremely typical wide-leaf individuals were collected to confirm the 194.2 kb region of QTL mapping using map-based cloned method, and the results showed the QTL interval is accurate (**Figure 6A**). Secondly, the 9 pairs of molecular markers were developed to screening the recombinant, and 9 useful recombinant individuals were identified. Finally, through comparing leaf width values, *qTLW4* was narrowed to an interval of 62.5 kb between two STS molecular markers qLW4-38 and qLW4-31 (**Figure 6B**). According to Rice Genome Annotation Project (http://rice.plantbiology.msu.edu/cgi-bin/gbrowse/rice/#search), a total of 8 genes were predicted in the region (**Figure 6C** and **Supplementary Table S4**).

### Analysis of Candidate Genes

**Figure 7:**
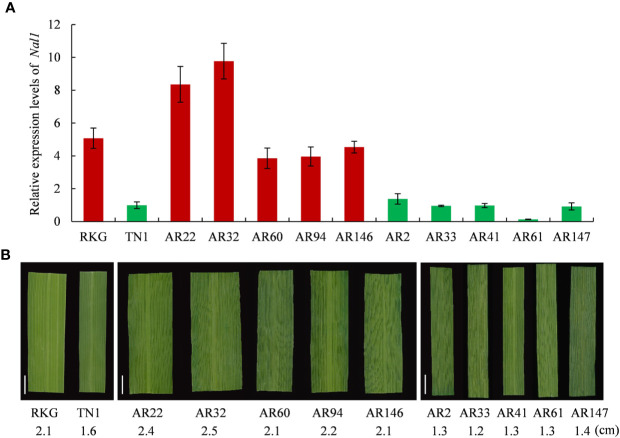
Expression analysis of *NAL1* and comparison analysis of leaf width. **(A)** Relative expression levels of *NAL1* in recombinant inbred line (RIL) parents, five wider leaf lines and five narrow leaf lines. **(B)** The corresponding leaf morphology and leaf width of **(A)**. Scale bar = 1cm.

Among the 8 genes, *LOC_Os04g52479* encodes a putative trypsin-like serine and cysteine proteases, which is the previously reported gene of *NAL1* (*Narrow Leaf 1*, Qi et al., 2008). Due to *NAL1* plays a positive role in regulating leaf width (Qi et al., 2008; Zhang et al., 2014), we carried out a quantitative reverse transcription PCR (qRT-PCR) to analyze the *NAL1* expression in two parental cultivars RKG, TN1, five wide-leaf lines and five narrow-leaf lines among RILs (**Figure 7**). The results indicated that the relative expressions of RKG and wide-leaf lines were significantly higher than TN1 and narrow-leaf lines (**Figure 7A**). So, we speculated that *NAL1* is the candidate gene of *qTLW4*.

## Discussion

In modern molecular breeding practice of rice cultivar, it is crucial to detect QTLs of important agronomic traits based on elite germplasm resources, and a high-quality genetic map with high-density uniformly distributed markers ensure an efficient tool for QTL mapping (Yu et al., 2011; Gao et al., 2013). However, limited number of traditional markers are difficult to cover the whole genome which affects the accuracy of QTL mapping, and whole-genome sequencing is costly and time-consuming. The rapid development of second-generation sequencing technologies provides an effective way to develop numerous valid markers to construct high-density genetic map in a short time. SLAF-seq technology combines site-specific amplification and high-throughput sequencing. Only specific DNA digested fragments need to be sequenced instead of whole genome sequencing, which greatly reduces the workload and cost (Sun et al., 2013). It has become an effective solution for large-scale genotyping and also has been successfully applied to the linkage map construction and QTL analysis in multiple species (Li et al., 2014; Zhu et al., 2016; Yin et al., 2018; Zhu et al., 2018; Wu et al., 2019; Yu et al., 2019).

In rice, the SLAFs are usually used to construct high-density linkage genetic map, carry out QTL mapping and association analysis (Mao et al., 2015; Chen et al., 2016; Peng et al., 2016; Zheng et al., 2016; Li et al., 2017; Yang et al., 2018). In the present study, we constructed a high-density linkage genetic map by a set of 149 RILs, which contained 10,760 valid SLAF markers with 0.17 cM average distance. Only two chromosomes exhibited max gap > 10 cM, and the distances were 10.03 cM and 11.89 cM on chromosome 1 and 7, respectively. In addition, spearman correlation coefficients of 12 linkage groups were 1 or close to 1, suggesting the good collinearity between genetic map and physical map, which revealed the high accuracy in genetic recombination rate. Also in rice, Mao et al. (2015) constructed a high-density genetic map spanned 1,537.1 cM in length with 0.32 cM intervals on average, and Peng et al. (2016) developed a SLAFs genetic map with 2,508.65 cM genetic length and 0.29 cM average distance. The average interval distance of two maps were both higher than 0.17cM, which indicated that the genetic map we constructed was denser and more suitable to conduct genetic analysis.

Due to the 10,760 molecular mark beyond the maximum calculating capacity in many QTL software, qgene software were finally used to conduct the QTL analysis, and a total of 24 QTLs involved in third leaf size were detected in Lingshui Hainan in 2017 and Hangzhou Zhejiang in 2017, 2018, respectively. In the result, 5 QTLs, *qTLLW1*, *qTLW3*, *qTLL4*, *qTLW4*, and *qTLW9* were repeatedly detected in three areas (**Figure 5**, **Table 5**, **Supplementary Figure S6**), including 3 leaf width related QTLs on chromosome 3, 4, and 9, 1 leaf length related QTL on chromosome 4, and 1 leaf length and width related QTLs on chromosome 1. Among them, *qTLLW1* was narrowed in a region of 1101.7 kb and explained phenotypic variance of 16.9% to 33.8%. Using a chromosome segment substitution lines (CSSLs) developed from Habataki and Sasanishiki, Bian et al. (2014) located a major QTL *qFLW1.3* for flag leaf width, which accounted for phenotypic variance of 25.45% and 31.4% in two area and exhibited an adjacent region as *qTLLW1*. However, no major QTL related leaf length was reported in the position of *qTLLW1*. Furthermore, another major QTL *qTLW4* accounting for 11.9%–25.3% phenotypic variance was also repeatedly detected in the same interval in Lingshui and Hangzhou, and had positive effect to increase leaf width. Using BC_2_F_2_ population, *qTLW4* was finally fine mapped in an interval of 62.5kb, in which contained *NAL1* gene. *NAL1* encodes a plant-specific protein involved in polar auxin transport and vascular patterns, and its loss-of-function mutation presented narrow leaf phenotype (Qi et al., 2008; Jiang et al., 2015). Moreover, several *NAL1* allelic types, including alternative splicing, amino acid substitutions and expression levels difference, were identified from natural variety which exhibited wide leaf phenotype and significantly increased yield (Chen et al., 2012; Fujita et al., 2013; Zhang et al., 2014). We therefore carried out an expression analysis of *NAL1*, and the results showed the expression in RKG and 5 wide leaf lines were significantly higher than TN1 and 5 narrow leaf lines. Combining the overexpression of *NAL1* gene significantly increased leaf width (Fujita et al., 2013; Zhang et al., 2014), so we speculated that *qTLW4* is an allele of the *NAL1* gene. It provides a new *NAL1* allele resource for genetics and breeders.

Leaf is an important vegetative organ, and its leaf shape and spatial posture directly affect photosynthesis, which in turn affects rice yield. In this study, we identified two major QTLs, *qTLW4* and *qTLLW1*, accounted for maximum 25.3% phenotypic variance in leaf width, and 33.8% and 22.5% in leaf length and width, respectively. Among them, *qTLLW1* exhibited positive additive effect on leaf length and negative effect on leaf width, indicated that the locus from RKG played a promoting effect on elongating leaf length and reducing leaf width. Another major QTL *qTLW4* presented positive additive effect on leaf width, also revealed that the segment of RKG had a positive effective in increasing leaf width. Therefore, the two QTLs, especially the new locus *qTLLW1*, are useful for facilitate breeding process of elite rice in ideal leaf type.

## Conclusion

In this study, a high-density genetic map with 10,760 SLAF markers was constructed, which spanned 1,613.59 cM genetic distance with 0.17 cM interval on average. Based on the map, a total of 24 QTLs underlying leaf morphology of third leaf were detected, accounted for a phenotypic variance ranged from 9% to 33.8%. Among them, two major QTLs, *qTLLW1* regulating both leaf length and width, and *qTLW4* only affecting leaf width were identified. Further fine mapping and expression analysis indicated that *qTLW4* may be the allelic gene to *NAL1*. The results laid a theoretical and material basis for rice molecular breeding of ideal plant architecture, especially in leaf morphology.

## Data Availability Statement

The raw sequencing data were uploaded to the NCBI (https://www.ncbi.nlm.nih.gov/sra/, accession number SRR12043868-SRR12044018).

## Author Contributions

Experimental design: JH and QQ. Map construction: YiW, YF, PH, YT, and YuW. Trait measurement and experiments: YiW, YF, LH, XD, HW, and LixZ. Data analysis: LiZ, GC, DZ, LG, GZ, ZG, GD, DR, LS, QZ, and DX. Manuscript preparation: YiW, YF, PH, QQ, and JH.

## Funding

This research was supported by the National GMO New Variety Breeding Program of PRC (2016ZX08011-001), the Zhejiang Province Outstanding Youth Fund (LR19C130001), the Natural Science Foundation of China (No. 31671666, 31871594, 31861143006), the National Science Foundation of Zhejiang Province (Y19C130005), and the Open Foundation of State Key Laboratory of Rice Biology (20190103).

## Conflict of Interest

The authors declare that the research was conducted in the absence of any commercial or financial relationships that could be construed as a potential conflict of interest.
