# Supplementary material for: Construction of a High-Density Genetic Map Based on SLAF Markers and QTL Analysis of Leaf Size in Rice
Source: Front Plant Sci. 2020 Jul 31;11:1143. doi: 10.3389/fpls.2020.01143 (PMC7411225; doi:10.3389/fpls.2020.01143)
Supplement: Supplementary file 1 [file Presentation_1.pdf]

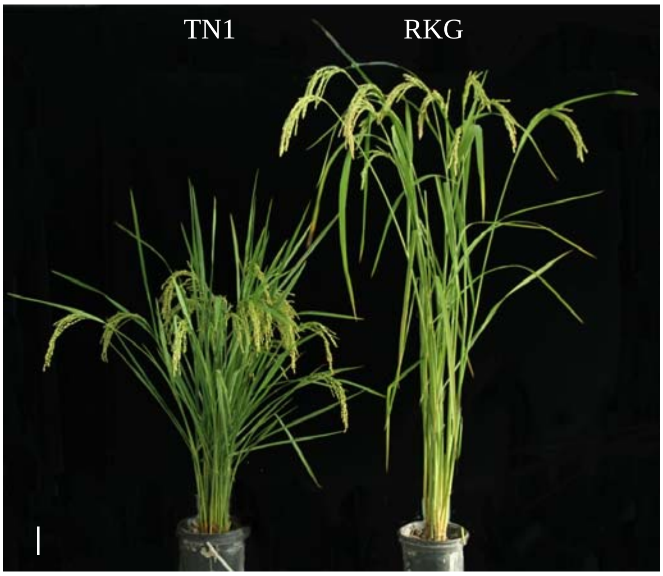

Supplementary Figure S1. Phenotype of TN1 and RKG.

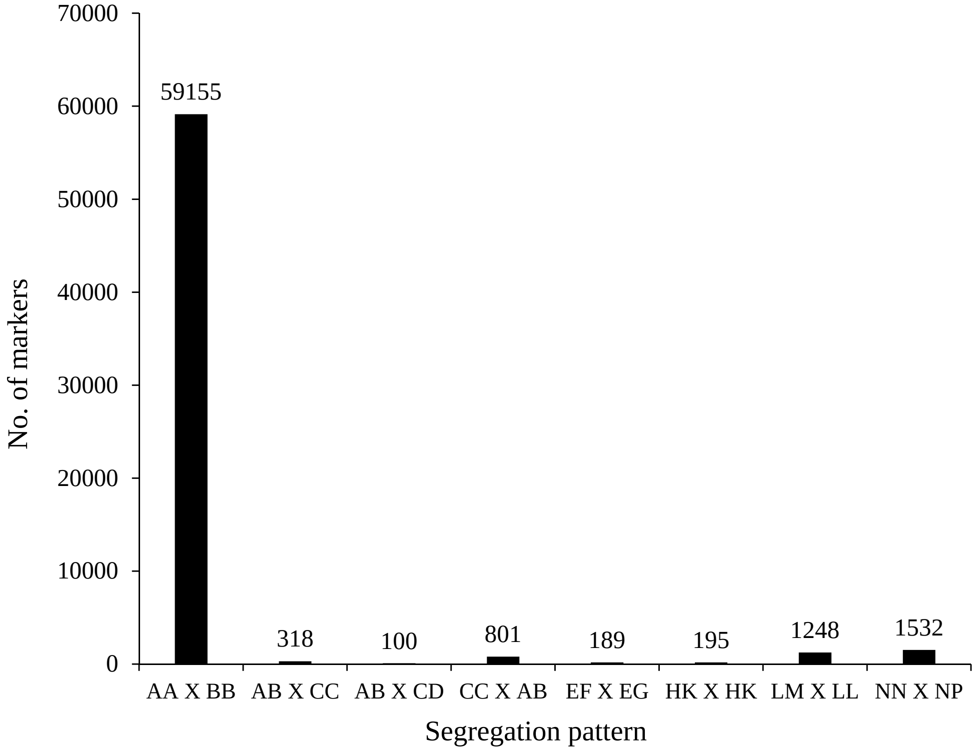

Supplementary Figure S2. Distribution of genotyped SLAF markers. The x-axes indicate the segregation pattern genotype of all SLAF markers; the y-axe indicate the numbers of SLAF markers.

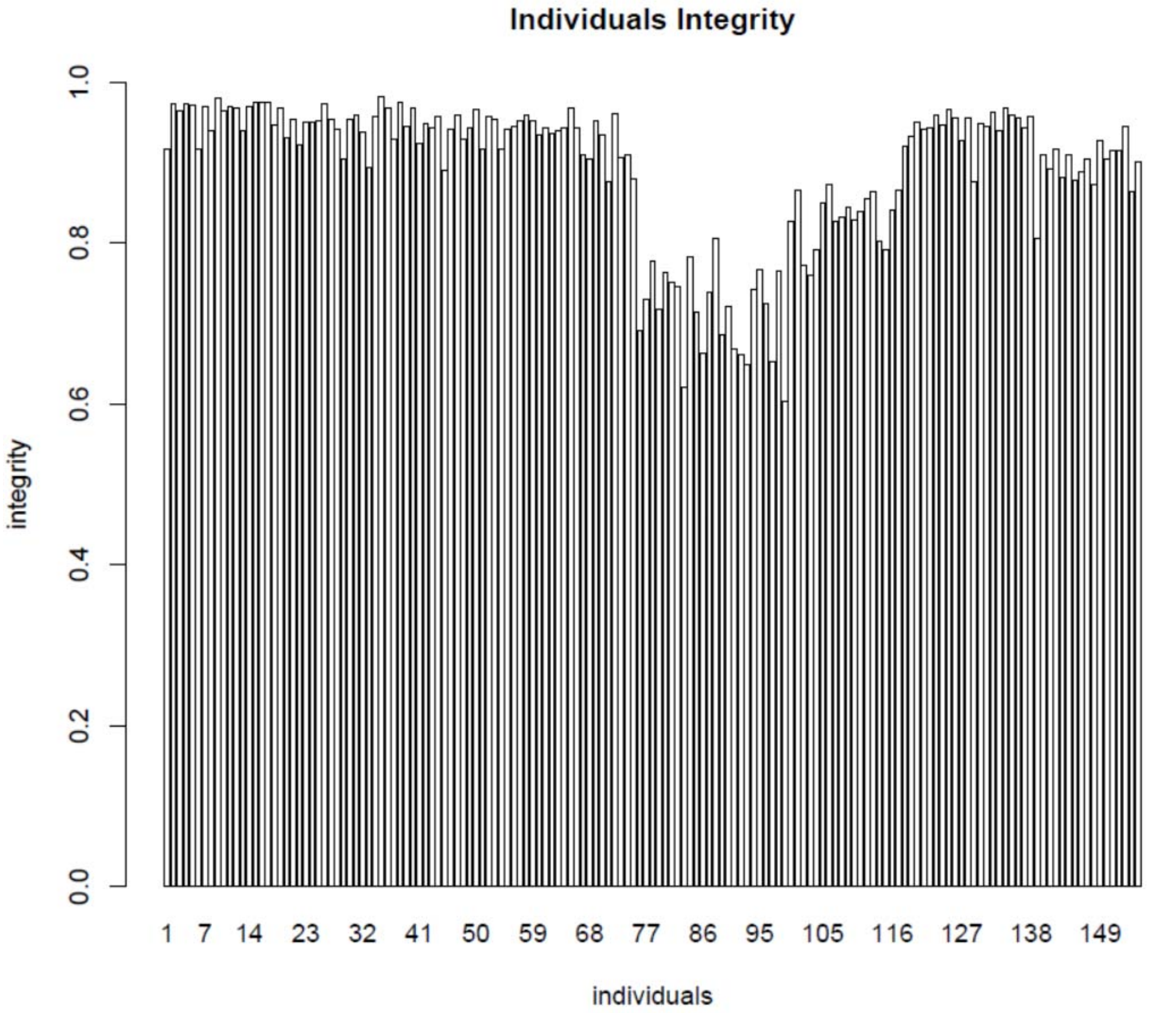

Supplementary Figure S3. The intergrity of the SLAF markers on the map of 149 individual in RILs.

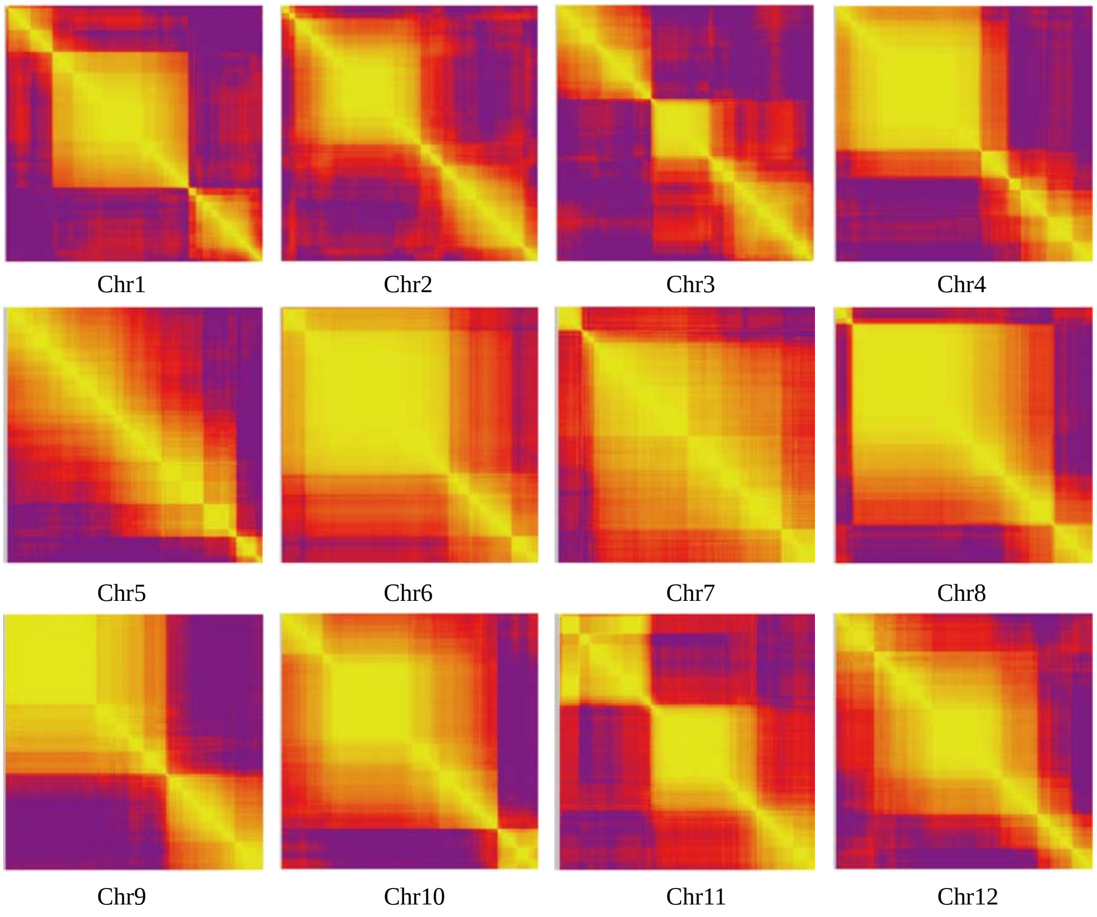

Supplementary figure S4. Heat maps of 12 linkage groups. Each row and column represents marker arrangement in the order of the genetic map. Each small square on the heat map represents the recombination rate between the adjacent markers. The color change from yellow to purple indicates the level of recombination rate changing from low to great. Yellow represents a lower recombination frequency means a closer distance between two adjacent markers, purple represents a greater recombination frequency means a further distance between two adjacent markers.

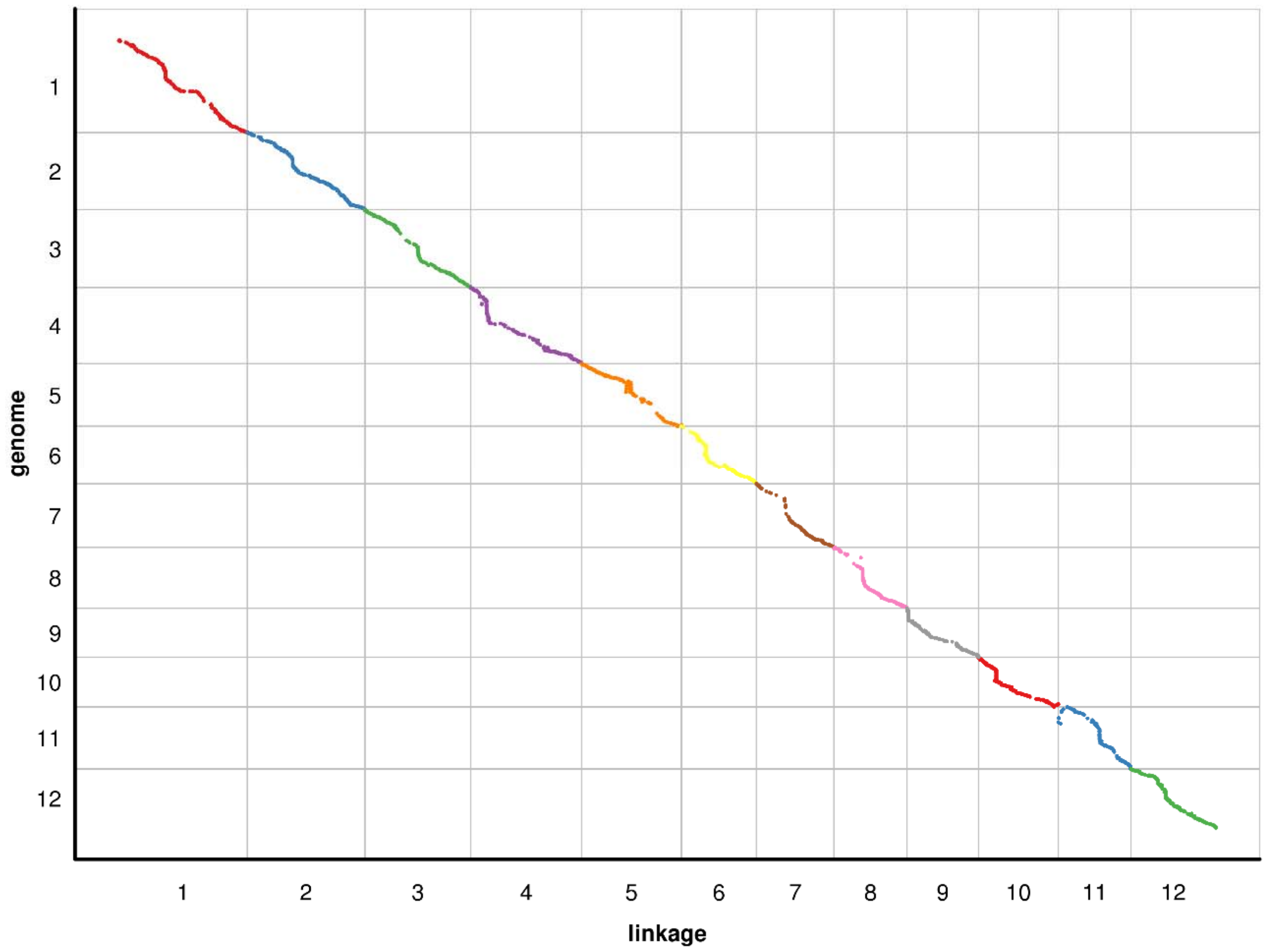

Supplementary Figure S5. The collinearity map of 12 linkage groups on genetic map with rice reference genome. The x-axes represents the genetic distance of each linkage group, the y-axe indicates the physical distance of each linkage group on the rice reference genome. Collinearity of all 10,760 SLAF markers are plotted as a scatter, different colors represent different LGs and chromosomes.

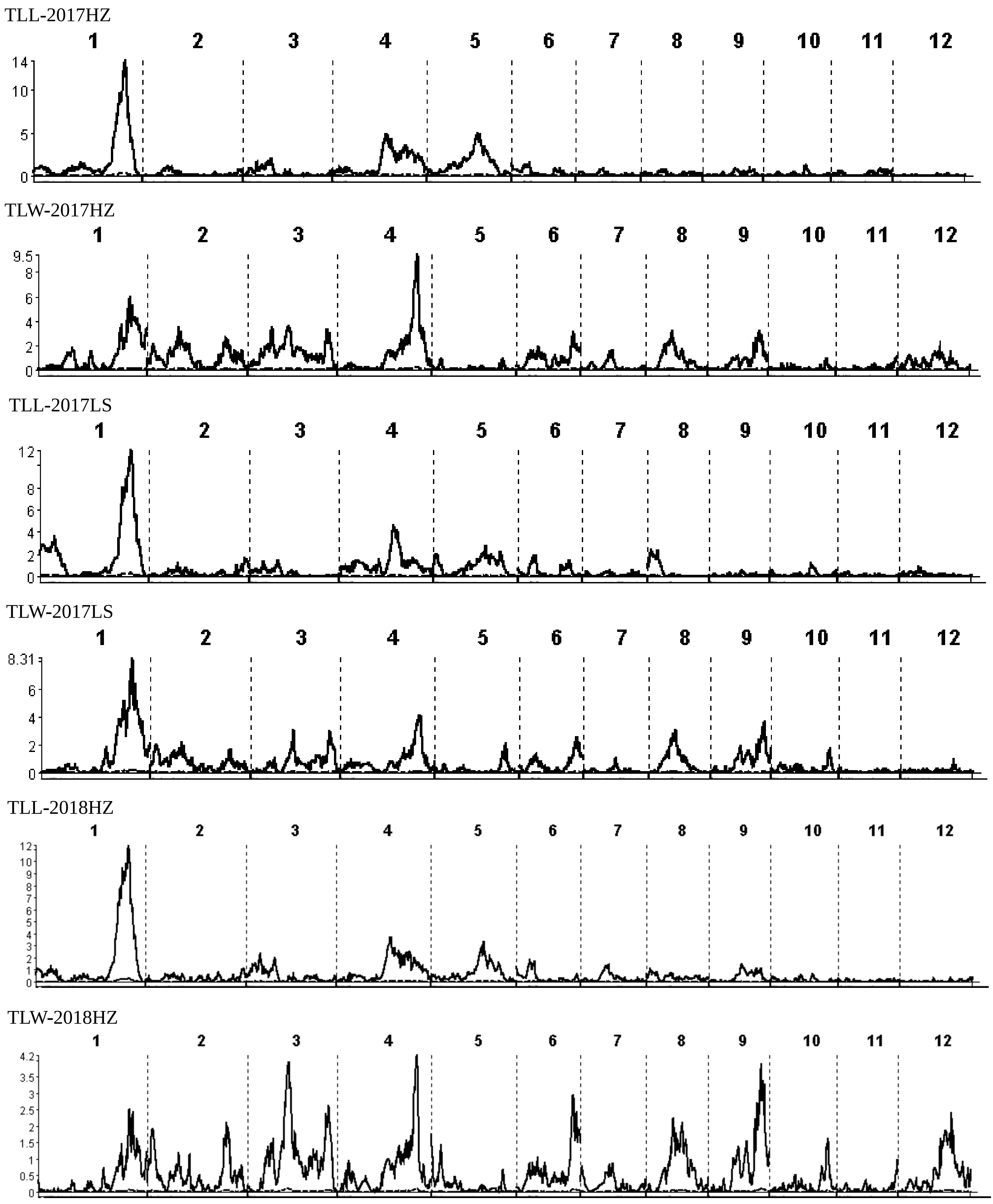

Supplementary Figure S6. Third leaf length and leaf width QTLs detected by Qgene software in 2017Hangzhou, 2017Lingshui and 2018Hangzhou.
