# Supplementary material for: Construction of a High-Density Genetic Map Based on SLAF Markers and QTL Analysis of Leaf Size in Rice
Source: Front Plant Sci. 2020 Jul 31;11:1143. doi: 10.3389/fpls.2020.01143 (PMC7411225; doi:10.3389/fpls.2020.01143)
Supplement: Supplementary file 2 [file Table_1.docx]

**Supplementary Table S1. Genotype coding rules**

| Segregation type | Paternal genotype | Maternal genotype | Offspring genotype |
| --- | --- | --- | --- |
| AB X CD | AB | CD | AC,AD,BC,BD,-- |
| EF X EG | EF | EG | EE,FE,EG,FG,-- |
| AB X CC | AB | CC | AC,BC,-- |
| CC X AB | CC | AB | AC,BC,-- |
| HK X HK | HK | HK | HH,HK,KK,-- |
| LM X LL | LM | LL | LM,LL,-- |
| NN X NP | NN | NP | NN,NP,-- |
| AA X BB | AA | BB | F_2_(AA,AB,BB),RIL/DH(AA,BB)-- |

--: genotype deletion in offspring.

**Supplementary Table S2. Primers used for transcripts analysis, sequencing and mapping**

| Marker | Forward primer sequence (5'-3') | Reverse primer sequence (5'-3') | Purpose |
| --- | --- | --- | --- |
| *NAL1*-RT | GCCCTTGAGTACAATGACGAGA | CAGGATAATAAGGCTTCCGCTA | QPCR |
| Actin | ACGGAGCGTGGTTACTCATTCA | CCTGTCCATCAGGAAGCTCGTA | QPCR |
| *NAL1*-promotor | GGCTGCACTTAAAATGGGATAGAGG | GCCTGCCACCTCTGAAGAAGTGAAG | Sequencing |
| *NAL1*-CDS | ATGAAGCCTTCGGACGATAAGGCGCA | TCATTTCTCCAGGTCAAGGCTTGATCCG | Sequencing |
| *NAL1*-C | ATGAAGCCTTCGGACGATAAGGCGC | TCATTTCTCCAGGTCAAGGCTTGATCC | Sequencing |
| qLW4-1 | CGCATCTCGCGCAGGTATGAAC | CAATCTCCCATCAATGGCGCCATG | Mapping |
| qLW4-2 | AGCCTATTATGCTTTAGGGAG | CTTGTTCTTAGCTGCTAAGCC | Mapping |
| qLW4-4 | CGTTGATACAAAGGCGATGCAC | CTTGGTATGCGCGATGGTTAC | Mapping |
| qLW4-6 | GTCTCCGGCAACCGCGTCGTCA | CGGTGAATCGCAAGAACAAACG | Mapping |
| qLW4-11 | ACATGTACAGCTGCCTCCCTC | GAGAACAGTGTGATCTTCAATG | Mapping |
| qLW4-38 | CTTGGCCAACAATTGTGGCATG | GTTTCCGCGGCAAGCCACCTG | Mapping |
| qLW4-31 | TCTAAATGGCTAAACATCGTG | AATATACGCGCTATCTAT | Mapping |
| qLW4-30 | ACTGGCTGGTTCATAACATGAGG | CTAAACGAGTGATCTGCAAGT | Mapping |
| qLW4-18 | CTAAACAACACTTGGAAGCGGCAACG | AGCTCTACCGGGGAGGGCGACAC | Mapping |

**Supplementary** **Table S3. Type of SLAFs**

| Type | Polymorphic SLAF | Non-Polymorphic SLAF | Repetitive SLAF | Total SLAF |
| --- | --- | --- | --- | --- |
| Number | 78,374 | 165,297 | 284 | 243,955 |
| Percentage | 32.13% | 67.76% | 0.12% | 100.00% |

The polymorphic site mainly includes SNPs and InDels.

**Supplementary** **Table S4.** The 62.5kb region including eight annotated genes

| Gene ID | Annotation |
| --- | --- |
| LOC_Os04g52450 | aminotransferase, putative, expressed |
| LOC_Os04g52460 | retrotransposon protein, putative, unclassified, expressed |
| LOC_Os04g52479 | peptidase, trypsin-like serine and cysteine proteases, putative, expressed |
| LOC_Os04g52500 | lecithine cholesterol acyltransferase, putative, expressed |
| LOC_Os04g52504 | adhesive/proline-rich protein, putative, expressed |
| LOC_Os04g52510 | glucosyltransferase, putative, expressed |
| LOC_Os04g52520 | APO, putative, expressed |
| LOC_Os04g52530 | heavy metal-associated domain containing protein, expressed |
